# Analysis of Collagen type X alpha 1 (COL10A1) expression and prognostic significance in gastric cancer based on bioinformatics

**DOI:** 10.1080/21655979.2020.1864912

**Published:** 2020-12-29

**Authors:** Shuai Chen, Yi Wei, Hanyang Liu, Yu Gong, Yan Zhou, Haojun Yang, Liming Tang

**Affiliations:** Center of Gastrointestinal Disease, The Affiliated Changzhou NO.2 People’s Hospital of Nanjing Medical University, Changzhou, China

**Keywords:** COL10A1, Collagen type X alpha 1, gastric cancer, biomarker, prognosis, bioinformatics

## Abstract

Collagen type X alpha 1 (COL10A1) is a member of the collagen family and the main matrix component. However, COL10A1 expression and prognosis relationship remains unclear in gastric cancer (GC). Through the analysis of database of Oncomine, the Cancer Genome Atlas (TCGA) as well as the Gene Expression Omnibus (GEO), in contrast to the tissue of normal gastric, COL10A1 in gastric cancer, had been upregulated. The high expression of COL10A1 was obviously related to T stage (*P* = 0.025) and lymph node metastasis (*P* = 0.025). It has been illustrated by the analysis of logistic regression that COL10A1’s heightened expression in gastric cancer had been essentially linked with pathological stage, tumor differentiation, and T classification. The Kaplan–Meier curve in the Kaplan-Meier plotter database (*P* = 0.0371) and GSE84437 (*P* = 0.002) indicate that patients with high COL10A1 expression possess poor prognosis, specifically GC patients with lymph node metastasis have it. TCGA’s Multivariate analysis (*P* = 0.025) and GSE84437 dataset (*P* = 0.034) show that high expression COL10A1 is a key independent predictor of poor overall survival. Searching KEGG pathway enrichment by GSEA, the results suggested that 29 pathways were enriched. qRT-PCR technique was used for verification of the COL10A1’s high expression in gastric cancer in contrast to the normal gastric tissues. In conclusion, COL10A1 is of great importance in predicting the survival rate of GC patients.

## Graphical abstract

**Figure:**
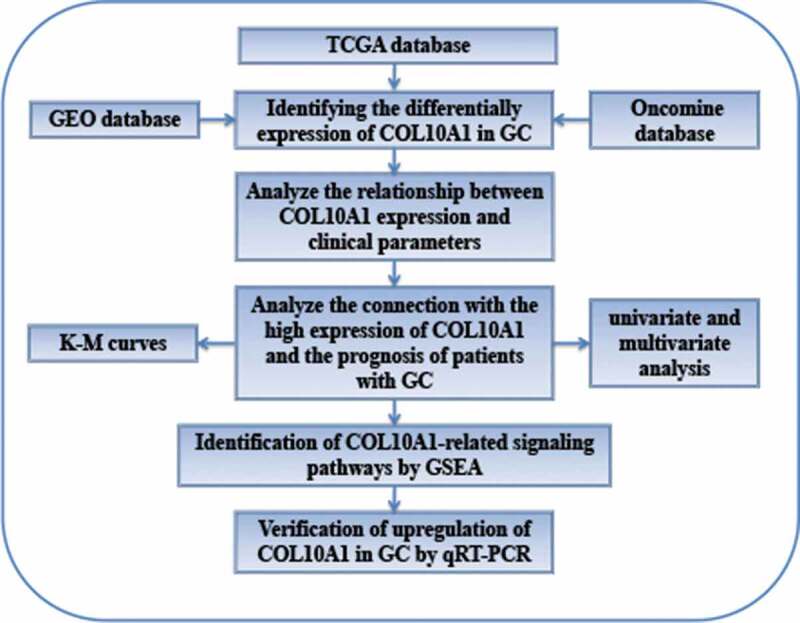

## Introduction

Gastric cancer (GC) is among the commonest malignant tumors related to the digestive system globally; also, GC comes as third major origin of deaths caused by cancer [1]. In GC, several patients have their diagnosis complete at the advanced stage [2]. Although progress has been made in the treatment of advanced gastric cancer, the clinical results of patients with advanced gastric cancer are still disappointing. Hence, identifying specific and sensitive biomarkers for the early diagnosis and prognosis evaluation for patients with GC is of great importance.

Collagen type X alpha 1 (COL10A1) comes from a family of collagen. COL10A1 gene; the alpha chain encoding form X collagen, which is the small chain collagen illustrated through hypertrophic chondrocytes during the procedure of endochondral ossification [3]. It is one primary matrix component in the stroma, and the extracellular matrix has been determined to play a significant role regarding growth, differentiation, progression, apoptosis and tumor cells’ metastasis [4]. Mutation of COL10A1 is associated with bowed leg stature caused by chondrodysplasia [5]. High expression of COL10A1 in various solid tumor tissues may be related to tumor angiogenesis [6]. Using the COL10A1 as a candidate biomarker, the occurrence of early cancer in gastric, colon, breast and lung cancer can be identified by serum detection [7–10]. The prognosis of patients is affected by the high expression levels in colorectal cancer tissue, and also, deemed as an independent factor of threat for overall survival rate [11]. However, it is still not clear that the correct mechanism and function of COL10A1 in the GC progression.

In this study, using Oncomine dataset, TCGA and GEO further explore the link shared by COL10A1 gene expression and patients’ clinicopathological characteristics with GC and its prognostic importance, so that additional proof for its potential function as a prognostic marker of gastric cancer can be given.

## Materials and methods

### TCGA data

The first quality mRNA information of 375 GC tissues and 32 nearby non-tumor tissues had been installed using the database of the Cancer Genome Atlas (https://portal.gdc.cancer.gov). The gastric patients’ clinical data have also been attained through the database of the Cancer Genome Atlas. Grade, age, sex, pathological stage, N stage, T stage, M stage and life status had been added in this database. Utilizing the Perl programming language to match the gene expression information with clinical information and to delete unknown or incomplete clinical information. The R software’s survival package had been utilized for analyzing the status of survival and gene expression.

### Microarray data

Through the GEO, the set of data was attained (https://www.ncbi.nlm.nih.gov/geo/). Expression data were extracted from five datasets (GSE26899, GSE103236, GSE2685, GSE29998 and GSE118916) and analyzed using GEO2R online. The relationship between COL10A1 expression and prognosis was verified by the dataset GSE84437 from the GEO database. GSE84437 contains clinical information (age, gender, T stage, N stage, survival status and survival time) of 433 patients with gastric cancer (Table 1).

**Table 1.** Characteristics of patients with gastric cancer in GSE84437 dataset

Characteristics	Variable	Patients (433)	Percentages (%)
Age	<65 years ≥65 years	267 166	61.66 38.34
Gender	Male Female	296 137	68.36 31.64
T classification	T1 T2 T3 T4	11 38 92 292	2.54 8.78 21.24 37.44
N classification	N0 N1 N2 N3	80 188 132 33	18.48 43.42 30.48 7.62
Vital status	Alive Death	224 209	51.73 48.27

### Oncomine analysis

In gastric cancer, the mRNA’s expression levels of gene as well as normal tissues were examined on the basis of the Oncomine platform (https://www.oncomine.org/resource/login.html). In this study, two-fold change, *P*-value = 1E-4 and top 10% gene rank were used as the threshold of our choice. The studies of Cui, Chi and D’Errico were used to analyze the differential expression levels of genes in GC.

### Databases of Kaplan–Meier plotter

Kaplan–Meier plots (https://kmplot.com/analysis/) combine the bigger amount of genes’ impact on the visualization of patients with cancer, through TCGA, EGA and GEO databases. It was utilized to assess the influence of the target gene on the prognosis of patients with gastric cancer, and the patients’ prognosis of having gastric cancer with different pathological parameters was analyzed by Kaplan–Meier plots.

### Gene set enrichment analysis

Preparation of expression data set file and phenotypic data file for single gene enrichment analysis of target gene by Perl software. Download and install GSEA (http://software.broadinstitute.org/gsea) software and Java8 runtime environment. The KEGG pathway enrichment analysis of the target gene was carried out, the path of the analysis comes from the c2.cp.kegg.v7.1.symbols.gmt data set in the MsigDB database. In GSEA, through the utilization of the weighted enrichment analysis technique, the enrichment examination had been conducted by random combination for 1000 times, and the *P* value and FDR value were calculated. And the results were visualized by R (plyr, ggplot2, grid, grid Extra package) software.

### Cell lines and clinical specimens

Gastric cancer cell lines (AGC, SGC-7901, MGC-803, BGC-823 and MKN-45) and normal gastric epithelial cells (GES-1) had been bought from the Cell Bank of the Chinese Academy of Sciences in Shanghai. Postoperative tissue samples from 30 patients with GC who were treated at the Changzhou No. 2 People’s Hospital from 2018 to 2019 were used in this study. In addition, tumor Para cancerous as well as tissues had been collected while the surgeries and were kept at −80°C immediately.

### Quantitative real-time polymerase chain reaction (qRT-PCR) analysis

In order to extract total RNA, the tissues and Cell lines had been pre-processed. Through the utilization of a Prime Script RT reagent kit (TaKaRa, Dalian, China) the cDNA was synthesized. Quantitative PCR was carried out with a 7500 real-time PCR system (ABI, Waltham, MA, USA), PCR primers had been synthesized through and bought from Sangon Biotech (Shanghai, China). COL10A1: forward: AAGAATGGCACCCCTGTAATGT, reverse: ACTCCCTGAAGCCTGATCCA; GAPDH: forward: CATGTTCCAATATGATTCCAC, reverse: CCTGGAAGATGGTGATG. GAPDH served as an internal control, and fold change had been calculated using the 2^−ΔΔ*CT*^ technique.

### Statistical analysis

Mann–Whitney U test had been utilized to analyze the differential articulation of COL10A1 in GC and normal tissues that are present in adjacent; The chi-square (ᵪ^2^) test had been utilized to analyze the link between high and COL10A1 expressions and clinicopathological features of patients; Kaplan-Meier method was used to draw the curve, Log-rank test was carried out to analyze the relationship between COL10A1 expression and OS in patients with gastric cancer, and Cox relapse was utilized to ascertain the danger proportion (Hazard ratio, HR) and its 95% certainty stretch (Confidence interval, CI) to break down its incentive in foreseeing the forecast of GC. All factual investigations were performed utilizing R programming (adaptation 3.6.3), and *P* < 0.05 was utilized to decide the noteworthiness level.

### Results

COL10A1, as an oncogene in gastric cancer, affects the occurrence and development of tumors and may be used as a new therapeutic target to improve the prognosis of patients with gastric cancer in the future. Through multiple databases and experiments, it is proved that COL10A1 plays a role as an oncogene in gastric cancer. Meanwhile, we found the relationship between COL10A1 and clinical parameters and prognosis of patients and further confirmed that COL10A1 is an independent factor that can predict the prognosis of patients by univariate and multivariate analysis. GSEA analysis shows that high expression of COL10A1 may regulate the progression of gastric cancer through multiple pathways.

### Differential expression of COL10A1 in gastric cancer

**Figure 1.**
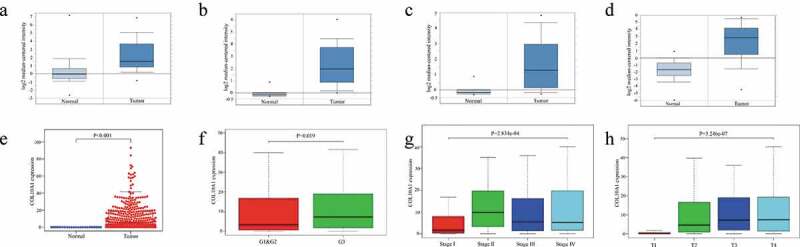
Expression level of COL10A1 in GC and its relationship with pathological parameters. The mRNA expression of COL10A1 in GC (a), diffuse gastric adenocarcinoma (b) and gastric intestinal-type adenocarcinoma (c, d) compared to normal individuals derived from the Oncomine database. Comparison of COL10A1 expression between GC tissues and adjacent nontumor tissue base on TCGA data (e). The expression of COL10A1 is grouped by tumor differentiation (f), pathological stage (g) and T stage (h)

The COL10A1 levels of expressions in GC had been analyzed using the Oncomine database (Supplementary Table 1). Higher expression of COL10A1 was observed in GC (Figure 1(a)), gastric adenocarcinoma in diffused state (referred in Figure 1(b)) and gastric intestinal type adenocarcinoma (Figure 1(c,d)) than in the corresponding normal tissues. Moreover, the same results were shown (Figure 1(e)) in TCGA data in the cancer related to gastric area (*P* < 0.001). Referring to mining of GEO database, we found that COL10A1 was profoundly communicated in GC tissues thought about with adjacent tissue that are in normal state in five datasets (GSE26899: logFC = 1.860, *P* < 0.01; GSE103236: logFC = 6.084, *P* < 0.001; GSE2685: logFC = 1.835, *P* < 0.05; GSE29998: logFC = 4.910, *P* < 0.001; GSE118916: logFC = 2.009, *P* < 0.01) (Supplementary Table 2).

### Relationship between expression of COL10A1 and clinicopathological parameters

The articulation levels of COL10A1 were diverse in bunches arranged by tumor differentiation (*P* = 0.019, Figure 1(f)), pathological stage (*P* < 0.001, Figure 1(f)) and T stage (*P* < 0.001, Figure 1(h)). Also, as per the articulation estimation of COL10A1, they were partitioned into two gatherings: a high-COL10A1 articulation gathering and a low-COL10A1 articulation gathering, as can be seen in Table 2. The high articulation level of COL10A1 was essentially identified with T stage (*P* = 0.025) and lymph node metastasis (*P* = 0.025). Logistic regression analysis demonstrated that the expanded articulation of COL10A1 in GC was fundamentally connected with tumor differentiation (OR = 1.543 for G3 versus G2 and G1, *P* = 0.043), pathological stage (OR = 3.106 for stage II versus stage I, *P* = 0.001; OR = 2.062 for stage III versus stage I, *P* = 0.031) and T stage (OR = 14.727 for T2 versus T1, *P* = 0.011; OR = 21.273 for T3 versus T1, *P* = 0.003; OR = 22 for T4 vs. T1, *P* = 0.003) (Table 3).

**Table 2.** Relationship between COL10A1 expression and clinicopathological parameters in gastric cancer

Clinicopathological parameters	COL10A1 expression			
	Hight (*n* = 159)	Low (*n* = 158)	Total	*P*-value
Age <65 ≥65	66(49.6) 93(50.5)	67(50.4) 91(49.5)		
Gender Male Female	102(51.5) 57(47.9)	96(48.5) 62(52.1)	198 119	0.611
Grade G1&G2 G3	50(43.5) 109(54.0)	65(56.5) 93(46.0)	115 202	0.093
Pathological stage I&II III&IV	75(51.7) 84(48.8)	70(48.3) 88(51.2)	145 172	0.689
T classifification T1–T2 T3–T4	31(38.8) 128(54.0)	49(61.2) 109(46.0)	80 237	0.025
Lymph node metastasis Negative Positive	55(54.5) 104(48.1)	46(45.5) 112(51.9)	101 216	0.025
Distant metastasis No Yes	148(50.0) 11(52.4)	148(50.0) 10(47.6)	296 21	1

**Table 3.** COL10A1 expression correlated with clinicopathological parameters

Clinicopathological parameters	Total (*N*)	Odds ratio in COL10A1 expression	*P*-value
Age ≥65 vs. <65	371	1.035(0.687–1.559)	0.870
Gender Male vs. female	375	1.088(0.713–1.662)	0.694
Tumor differentiation G3 vs. G2&G1	366	1.543(1.015–2.356)	0.043
Pathological stage Stage II vs. Stage I Stage III vs. Stage I Stage IV vs. Stage I	164 203 91	3.106 (1.577–6.307) 2.062(1.079–4.062) 2.118(0.902–5.061)	0.001 0.031 0.086
T classification T2 vs. T1 T3 vs. T1 T4 vs. T1	99 187 119	14.727(2.827–271.308) 21.273(4.243–387.099) 22(4.286–403.417)	0.011 0.003 0.003
Lymph node metastasis Positive vs. negative	357	0.870(0.555–1.363)	0.543
Distant metastasis Yes vs. no	355	0.923(0.404–2.094)	0.847

### Prognostic significance of COL10A1 expression in GC

**Figure 2.**
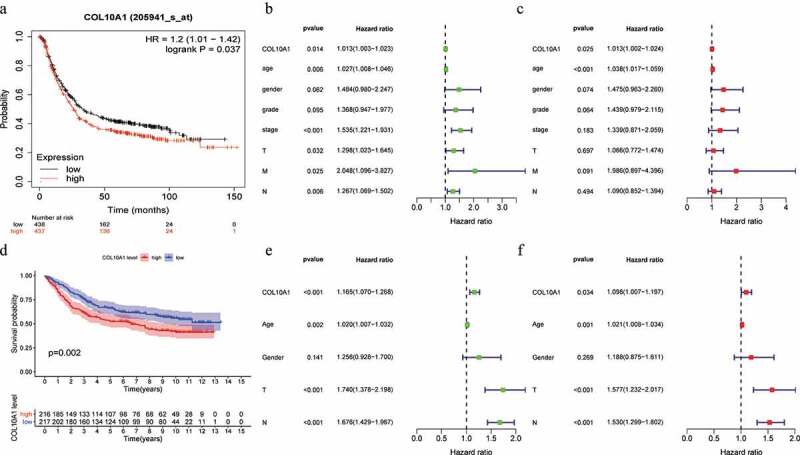
Survival analysis, univariate and multivariate analysis. Kaplan-Meier curve of the relationship between COL10A1 mRNA expression and the prognosis of GC patients based on Kaplan-Meier plots database (a) and GSE84437 dataset (d). Univariate and multivariate analysis of COL10A1 expression and its correlation in patients with GC base on TCGA data (b, c) and GSE84437 dataset (e, f)

The databases in Kaplan–Meier plotter were used to survey the relationship within the expression of COL10A1 as well as prognosis in GC. High COL10A1 expression was associated with unfavorable prognosis in GC (OS: HR = 1.2, 95% CI = 1.01–1.42, *P* = 0.0371; Figure 2(a)). Kaplan–Meier risk estimates were used to assess the guess of 433 GC patients with COL10A1 expression in GSE84437 dataset, the results verified that high COL10A1 expression was more obvious with poor generally endurance than low COL10A1 articulation (*P* = 0.002, Figure 2(d)).

Using the databases of Kaplan–Meier Plotter, it is also analyzed that the effect of COL10A1 expression on the aspects of patients with different clinical types (Table 4). High COL10A1 expression correlated with both poorer OS and PPS in stage III patients (OS: HR = 1.90, *P* = 1.7e-05; PPS: HR = 2.6, *P* = 3E-05), stage T3 patients (OS: HR = 1.76, *P* = 0.001; PPS: HR = 1.66, *P* = 0.0099), stage M0 patients (OS: HR = 1.74, *P* = 0.00015; PPS: HR = 1.51, *P* = 0.0068), intestinal patients (OS: HR = 2.20, *P* = 9.4e-07; PPS: HR = 1.66, *P* = 0.0161), diffuse patients (OS: HR = 1.66, *P* = 0.0049; PPS: HR = 1.67, *P* = 0.021) and HER2 positive patients (OS: HR = 1.74, *P* = 8.6e-05; PPS: HR = 1.53, *P* = 0.03). Specifically, high expression of COL10A1 affects the prognosis of patients with lymph node metastasis (OS: stage N1, HR = 1.97, *P* = 0.0012; stage N2, HR = 2.78, *P* = 0.00016; stage N3, HR = 1.79, *P* = 0.035; stage N1 + 2 + 3, HR = 1.82, *P* = 6.6e-06. PPS: stage N1, HR = 1.61, *P* = 0.039; stage N2, HR = 2.17, *P* = 0.016; stage N1 + 2 + 3, HR = 1.6, *P* = 0.0011). However, COL10A1 expression did not correlate with OS and PPS in stage II, stage T2, stage N0, stage M1, and HER2 negative patients. These data show that prognostic significance of COL10A1 expression in GC patients based on their clinical characteristics, usually in patients with lymph node metastasis of gastric cancer.

**Table 4.** Correlation of COL10A1 mRNA expression and clinical prognosis in gastric cancer

	Overall survival	Post progression survival
	N HR *p-*Value	N HR *p-*Value
Sex Female Male	236 1.94(1.32–2.77) 0.00047 544 1.35(1.06–1.71) 0.013	149 1.48(0.95–2.29) 0.081 348 1.27(0.98–1.64) 0.074
Stage 1 2 3 4	69 0.11(0.02–0.49) 0.00046 140 0.65(0.34–1.23) 0.18 305 1.90(1.41–2.57) 1.7e-05 148 1.82(1.23–2.96) 0.0023	31 0.08(0.01–0.72) 0.0051 105 0.71(0.37–1.38) 0.31 142 2.6(1.63–4.14) 3.2e-05 104 1.45(0.89–2.38) 0.13
Stage T 1 2 3 4	14 - - 241 1.36(0.87–2.12) 0.18 204 1.76(1.25–2.49) 0.0011 38 0.56(0.24–1.35) 0.19	3 - - 196 1.25(0.79–1.99) 0.33 150 1.66(1.12–2.44) 0.0099 29 - -
Stage N 0 1 2 3 1 + 2 + 3	76 0.49(0.21–1.12) 0.085 225 1.97(1.30–3.00) 0.0012 121 2.78(1.60–4.85) 0.00016 76 1.79(1.03–3.09) 0.035 422 1.82(1.40–2.37) 6.6e-06	41 0.35(0.11–1.09) 0.059 169 1.61(1.02–2.53) 0.039 105 2.17(1.33–3.56) 0.0016 63 1.6(0.85–2.99) 0.14 337 1.6(1.2–2.13) 0.0011
Stage M 0 1	444 1.74(1.30–2.33) 0.00015 56 1.96(1.05–3.67) 0.33	342 1.51(1.12–2.04) 0.0068 36 2.35(1.04–5.33) 0.035
Lauren classification Intestinal Diffuse Mixed	320 2.20(1.59–3.04) 9.4e-07 241 1.66(1.16–2.36) 0.0049 32 – -	192 1.66(1.09–2.51) 0.0161 176 1.67(1.07–2.58) 0.021 16 – -
Differentiation Poorly differentiated Moderately differentiated Well differentiated	165 2.08(1.27–3.40) 0.0029 67 2.34(1.21–4.53) 0.0098 32 – -	49 1.36(0.72–2.58) 0.3428 24 – - 0 – -
HER2 status HER2 negative HER2 positive	532 1.22(0.96–1.56) 0.11 343 1.74(1.31–2.30) 8.6e-05	334 1.19(0.89–1.59) 0.24 164 1.53(1.04–2.26) 0.03

### The influence of COL10A1 articulation on endurance by univariate and multivariate investigation

Univariate investigation concluded that COL10A1 expression (HR = 1.013, 95% CI = 1.003–1.023, *P* = 0.014), age (HR = 1.027, 95% CI = 1.008–1.046, *P = *0.006), pathological stage (HR = 1.535, 95% CI = 1.221–1.931, *P* < 0.001), T stage (HR = 1.298, 95% CI = 1.023–1.645, *P* = 0.032), M stage (HR = 2.048, 95% CI = 1.096–3.827, *P* = 0.025) and N stage (HR = 1.267, 95% CI = 1.069–1.502, *P* = 0.006) are significant predictors to predict the survival chance (Figure 2(b)). The declaration of COL10A1 and other clinical data (counting age, sex, grade, pathological stage, T stage, M stage and N stage) were remembered for the multivariate examination. The outcomes demonstrated that the high articulation of COL10A1 was a significant autonomous indicator of helpless by and large endurance (HR = 1.013, 95% CI = 1.002–1024, *P* = 0.025) (Figure 2(c)). In addition, using univariate and multivariate analysis in GSE84437 dataset, COL10A1 was confirmed again as a significant autonomous indicator of helpless in general endurance (HR = 1.098, 95% CI = 1.007–1.197, *P* = 0.034) (Figure 2(e,f)).

### Recognition of COL10A1- associated signaling pathways by GSEA

**Figure 3.**
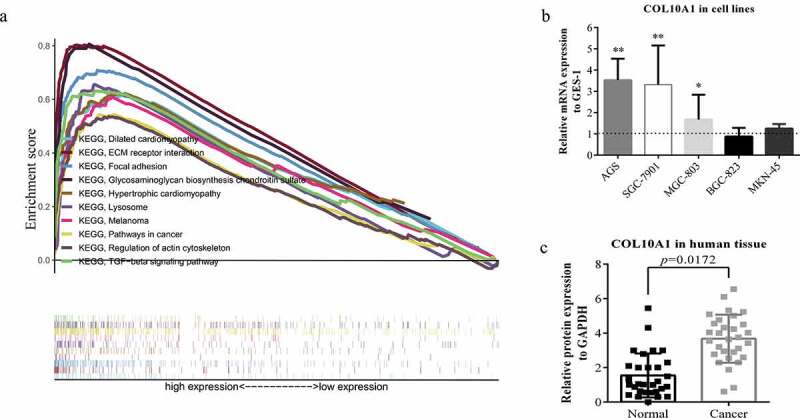
GSEA analysis and experimental verification. (a) A combined enrichment plot has been obtained from the analysis of the enrichment of the gene series, including the enrichment fraction and gene series (top 10 terms). (b) The expression level of COL10A1 was higher than that of GES-1 in three kinds of GC cells (AGS, SGC-7901 and MGC-803) (*P < 0.05, **P < 0.01). (c) qRT-PCR analysis of COL10A1 mRNA expression in 30 pairs of GC tissues and adjacent nontumor tissues

In light of TCGA information, the capacity of looking through COL10A1 and its related sign transmission was performed through GSEA. In the perspective on NES, FDR *q-*value, and nominal *p*-value, fundamentally advanced flagging pathways were chosen. In this study, 29 signaling measures were differentially enhanced in the profoundly communicated phenotypes of COL10A1: these were involved in focal adhesion, ECM receptor interaction, melanoma, TGF-β signaling, glycosaminoglycan biosynthesis chondroitin sulfate, hypertrophic cardiomyopathy, dilated cardiomyopathy, lysosome, regulation of actin cytoskeleton, pathways in cancer, glycosaminoglycan degradation, hedgehog signaling, basal cell carcinoma, glycosphingolipid biosynthesis, ganglio-series gangliosides, bladder cancer, cell adhesion molecules, cytokine-cytokine receptor interaction, axon guidance, Toll-like receptor signaling, notch signaling, renal cell carcinoma, GAP junctions, systemic lupus erythematosus, arrhythmogenic right ventricular cardiomyopathy, prion diseases, leishmania infection, pathogenic *Escherichia coli* infection, pancreatic cancer and leukocyte transendothelial migration (Supplementary Table 3, Figure 3(a)).

### Verification of upregulation of COL10A1 in GC by qRT-PCR

To additionally confirm that the articulation level of COL10A1 in GC tissues was higher than that in paracancerous tissues, we previously checked this in five GC cell lines (AGS, SGC-7901, MGC-803, BGC-823 and MKN-45) and gastric epithelial cells (GSE-1) (Figure 3(b)). The articulation enunciation levels of COL10A1 in AGS (*P* < 0.01), SGC-7901 (*P* < 0.01) and MGC-803 (*P* < 0.05) were likewise also higher than that in gastric epithelial cells. Using qRT-PCR technology to recognize the articulation verbalization of COL10A1 at the transcriptional level and found that the articulation explanation levels of COL10A1 mRNA in GC tissues were fundamentally higher than that in had been basically more noteworthy when contrasted with the neighboring non-tumor tissues (*P* = 0.0172, Figure 3(c)).

## Discussion

Gastric cancer is a tumor originating from the most superficial epithelial cells of the gastric wall. The occurrence of GC is a progressive process with the participation of many steps and factors. GC involves the influence of environment and dietary factors, *Helicobacter pylori* infection, genetic factors, smoking and drinking [12–16]. The early diagnosis of GC mainly depends on endoscopic screening [17]. An effective biomarker for the diagnosis of the GC is urgently needed.

In this study, through the Oncomine database, TCGA database and GEO database analysis, a comparison was made between high expression levels of COL10A1 in gastric cancer tissues and the adjacent normal tissues, which had been persistent with the outcomes of experimental verification and relevant research reports [18,19]. Meanwhile, it was independently reported in colorectal cancer, lung cancer and oral cancer, and the expression of COL10A1 gene in tumor tissue was higher than that in normal corresponding tissue [11,20,21]. It has been suggested by the outcomes that COL10A1 might be an oncogene and might have a vital function in the occurrence and development of tumors. In addition, in GC patients, COL10A1 expression levels had been distinct in groups classified as per the pathological stage, tumor differentiation and T stage. On this premise, the connection between the statement of COL10A1 and clinicopathological boundaries was additionally examined, and it was discovered that high articulation levels of COL10A1 were fundamentally related to T stage and lymph node metastasis. Besides, the high articulation of COL10A1 was identified with helpless forecast of patients with lymph node metastasis, while that of patients with low articulation of COL10A was better. Univariate examination shows that high COL10A1 articulation was identified with more regrettable OS. Clinicopathological boundaries, for example, pathological stage, T stage, N stage and M stage were related to the anticipation of patients with GC. The results show that COL10A1 is an independent prognostic factor for the survival of patients with GC, which proves that it may become a biomarker of GC. Using GSEA to analyze the signaling pathway of COL10A1 in GC, abiomarker of GC. Using GSEA to analyze the signaling pathway of COL10A1 in GC, a total of 29 signaling pathways were enriched. Among them, melanoma, pathway in cancer, basal cell carcinoma, bladder cancer, renal cell carcinoma and pancreatic cancer prove that COL10A1 affects the occurrence and development of cancer. It has been found that focal adhesion affects cell migration [22,23]. It was reported that focal adhesion was closely related to a number of biological pathways, which includes proliferation of cells, differentiation of cells and survival of cells [24]; it also affects the invasion of cancer cells [25]. ECM receptor interaction plays a very important role in the tumor microenvironment. A study showed that the extracellular matrix protein (ECM) in serum and tissue of patients with GC regulates the metastasis of GC cell and metabolism of glucose through ITGB4/FAK/SOX2/HIF-1α signaling process induced by ECM receptor interaction, which is of great significance for the development of therapeutic targets for the prevention of tumor metastasis and recurrence [26]. Li’s study found that COL10A1 promotes migration of GC cell and also its invasion through positive transcription regulation of SOX9 and participation in the transforming growth TGF-β signaling pathway [18]. Lysosomes are related to many diseases and tumor metastasis and drug resistance; inhibition of lysosome can overcome the chemotherapy resistance of some tumors and improve the efficacy of immunotherapy [27,28]. Regulation of actin cytoskeleton is correlated with migration and invasion of cancer [29], kinesin superfamily protein 2A, a key protein in this signaling pathway, playing the role of oncogenes in a variety of cancers [30–32]. The Hedgehog signaling processes play a significant part in the development of chronic gastritis to GC [33]; it also promotes the growth of GC cells [34] and improves the ability of migration and invasion [35]. The CAM pathway is related to tumor angiogenesis, invasion and metastasis [36]. Takashi’s results suggest that the expression of L1 cell adhesion molecule (L1CAM) may be used as an important biomarker for identifying high-risk patients with poor prognosis and as a therapeutic target in GC [37]. Cytokine-cytokine receptor interactions are important immune signaling pathways that regulate the occurrence and progression of cancer by regulating the interaction of cytokines [38]. Axon guidance has been reported to be involved in the occurrence and development of tumors [39]. Semaphorins and their receptors are significant axon guidance factors that participate in tumor cell migration [39]. The Toll-like receptor signaling pathway is critical for gastric cancer cell migration and proliferation [40,41]. The Notch signaling pathway is a crucial pathway in the occurrence and development of tumor [42]. Notch signal plays a significant part in the regulation of proliferation, invasion and apoptosis of GC cell [42–44]. It has been found that, in the formation of most tumors, the function of GAP junction is often decreased or eliminated, and the restoration of GAP junction of tumor cells can hinder the development and differentiation of tumor cells [45]. In the treatment of tumors, GAP junctions can increase the efficacy of a variety of antineoplastic drugs [46]. Other undiscussed signaling pathways may indicate that the COL10A1 gene is also involved in the regulation of non-tumor diseases. To summarize, COL10A1 encourages gastric cancer development by controlling several signaling pathways.

There exist few deficiencies as well as limitations in our study as well. First, the clinical data is not absolute and is lacking specific data on surgery, chemotherapy and tumor size. Second, the study is based on data from public databases and published articles, and the quality of data may affect the results. Third, the accuracy of the database used to analyze data and the choice of statistical methods may affect the interpretation of the research results. However, we obtained similar results by analyzing multiple databases and experimental confirmation, which supports our research conclusion.

## Conclusion

Our study confirmed that COL10A1 mRNA expression levels in GC tissues had been more as compared to the normal gastric tissues, as verified by experiments. High COL10A1 expression had been linked to the poor gastric cancer prognosis. The elevation of COL10A1 was linked to few clinic pathological features of gastric cancer. Meanwhile, it must be paid attention to that high expression levels of COL10A1 significantly affected the GC patients’ prognosis of having lymph node metastasis. It has been displayed by the Univariate and multivariate survival analysis that the upregulated expression of COL10A1 in gastric cancer happened to be the independent risk factor for shorter OS. These results suggest that the COL10A1 level of expression might be an index for the prognosis as well as diagnosis of gastric cancer. In future analysis, to determine the prognostic value of COL10A1 in GC, other clinical trials are required for the verification of the respective outcomes.

## Supplementary Material

Supplemental Material

## Data Availability

Data were analyzed from GEO (http://www.ncbi.nlm.nih.gov/geo), TCGA (https://portal.gdc.cancer.gov), Oncomine database (https://www.oncomine.org/resource/login.html) and Kaplan–Meier plots (https://kmplot.com/analysis/). We declare that the data and materials in this study will be provided free of charge to scientists for noncommercial purposes.

## Highlight

1. The role of COL10A1 as an oncogene in gastric cancer.
2. The expression of COL10A1 can be used to guide the prognosis of gastric cancer patients with lymph node metastasis.
3. High expression of COL10A1 is an independent risk factor for predicting the prognosis of patients with gastric cancer.
