# Supplementary figures and images for: Analysis of Collagen type X alpha 1 (COL10A1) expression and prognostic significance in gastric cancer based on bioinformatics
Source: Bioengineered. 2020 Dec 29;12(1):127–37. doi: 10.1080/21655979.2020.1864912 (PMC8291830; doi:10.1080/21655979.2020.1864912)

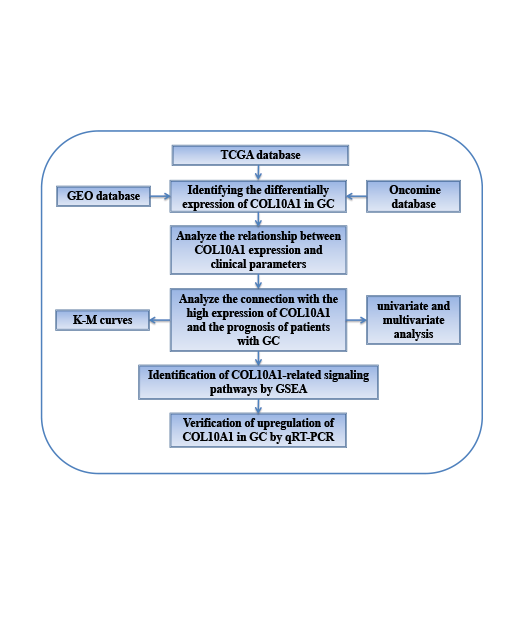

Supplement: Supplemental Material [file KBIE_A_1864912_SM9693.zip › supplement/GraphicalAbstract.png]
