# Supplementary material for: Analysis of Collagen type X alpha 1 (COL10A1) expression and prognostic significance in gastric cancer based on bioinformatics
Source: Bioengineered. 2020 Dec 29;12(1):127–37. doi: 10.1080/21655979.2020.1864912 (PMC8291830; doi:10.1080/21655979.2020.1864912)
Supplement: Supplemental Material [file KBIE_A_1864912_SM9693.zip › supplement/Supplementary Table 1.doc]

| Table 1. Characteristics of patients with gastric cancer in GSE84437 dataset. | | | |
| --- | --- | --- | --- |
| Characteristics | Variable | Patients (433) | Percentages(%) |
| Age | <65 years  ≥65years | 267  166 | 61.66  38.34 |
| Gender | Male  Female | 296  137 | 68.36  31.64 |
| T classification | T1  T2  T3  T4 | 11  38  92  292 | 2.54  8.78  21.24  37.44 |
| N classification | N0  N1  N2  N3 | 80  188  132  33 | 18.48  43.42  30.48  7.62 |
| Vital status | Alive  Death | 224  209 | 51.73  48.27 |
