# Supplementary material for: Analysis of Collagen type X alpha 1 (COL10A1) expression and prognostic significance in gastric cancer based on bioinformatics
Source: Bioengineered. 2020 Dec 29;12(1):127–37. doi: 10.1080/21655979.2020.1864912 (PMC8291830; doi:10.1080/21655979.2020.1864912)
Supplement: Supplemental Material [file KBIE_A_1864912_SM9693.zip › supplement/Supplementary Table 2.doc]

| Table 2. Relationship between COL10A1 expression and clinicopathological parameters in gastric cancer. | | | | |
| --- | --- | --- | --- | --- |
| Clinicopathological parameters | COL10A1 expression | | Total | *P*-value |
|  | Hight (n=159) | Low (n=158) |  |  |
| Age  <65  ≥65 | 66(49.6)  93(50.5) | 67(50.4)  91(49.5) | 133  184 | 0.961 |
| Gender  Male  Female | 102(51.5)  57(47.9) | 96(48.5)  62(52.1) | 198  119 | 0.611 |
| Grade  G1&G2  G3 | 50(43.5)  109(54.0) | 65(56.5)  93(46.0) | 115  202 | 0.093 |
| Pathological stage  I&II  III&IV | 75(51.7)  84(48.8) | 70(48.3)  88(51.2) | 145  172 | 0.689 |
| T classifification  T1–T2  T3–T4 | 31(38.8)  128(54.0) | 49(61.2)  109(46.0) | 80  237 | 0.025 |
| Lymph node metastasis  Negative  Positive | 55(54.5)  104(48.1) | 46(45.5)  112(51.9) | 101  216 | 0.025 |
| Distant metastasis  No  Yes | 148(50.0)  11(52.4) | 148(50.0)  10(47.6) | 296  21 | 1 |
