# Supplementary material for: Analysis of Collagen type X alpha 1 (COL10A1) expression and prognostic significance in gastric cancer based on bioinformatics
Source: Bioengineered. 2020 Dec 29;12(1):127–37. doi: 10.1080/21655979.2020.1864912 (PMC8291830; doi:10.1080/21655979.2020.1864912)
Supplement: Supplemental Material [file KBIE_A_1864912_SM9693.zip › supplement/Supplementary Table 3.doc]

| Table 3. COL10A1 expression correlated with clinicopathological parameters. | | | |
| --- | --- | --- | --- |
| Clinicopathological parameters | Total (*N*) | Odds ratio in COL10A1 expression | *p*-value |
| Age  ≥65 vs. <65 | 371 | 1.035(0.687-1.559 ) | 0.870 |
| Gender  Male vs. female | 375 | 1.088(0.713-1.662) | 0.694 |
| Tumor differentiation  G3 vs. G2&G1 | 366 | 1.543(1.015-2.356) | 0.043 |
| Pathological stage  Stage II vs. Stage I  Stage III vs. Stage I  Stage IV vs. Stage I | 164  203  91 | 3.106 (1.577-6.307)  2.062(1.079-4.062)  2.118(0.902-5.061) | 0.001  0.031  0.086 |
| T classification  T2 vs. T1  T3 vs. T1  T4 vs. T1 | 99  187  119 | 14.727(2.827-271.308)  21.273(4.243-387.099)  22(4.286-403.417) | 0.011  0.003  0.003 |
| Lymph node metastasis  Positive vs. negative | 357 | 0.870(0.555-1.363) | 0.543 |
| Distant metastasis  Yes vs. no | 355 | 0.923(0.404-2.094) | 0.847 |
